# Good Tumor Response to Chemoradioimmunotherapy in dMMR/MSI-H Advanced Colorectal Cancer: A Case Series

**DOI:** 10.3389/fimmu.2021.784336

**Published:** 2021-12-15

**Authors:** Chengjing Zhou, Ting Jiang, Yajie Xiao, Qiaoxuan Wang, Zhifan Zeng, Peiqiang Cai, Yongtian Zhao, Zhikun Zhao, Dongfang Wu, Hanqing Lin, Chao Sun, Rong Zhang, Weiwei Xiao, Yuanhong Gao

**Affiliations:** ^1^ Department of Radiation Oncology, State Key Laboratory of Oncology in South China, Collaborative Innovation Center for Cancer Medicine, Sun Yat-sen University Cancer Center, Guangzhou, China; ^2^ School of Medicine, Southern University of Science and Technology, Shenzhen, China; ^3^ Department of Medicine, YuceBio Technology Co., Ltd., Shenzhen, China; ^4^ Department of Medical Imaging and Interventional Radiology, State Key Laboratory of Oncology in South China, Collaborative Innovation Center for Cancer Medicine, Sun Yat-sen University Cancer Center, Guangzhou, China; ^5^ Department of Endoscopy and Laser, State Key Laboratory of Oncology in South China, Collaborative Innovation Center for Cancer Medicine, Sun Yat-sen University Cancer Center, Guangzhou, China

**Keywords:** chemotherapy, radiotherapy, immunotherapy, programmed cell death protein 1 inhibitor, colorectal cancer

## Abstract

**Purpose:**

Immune checkpoint blockade has led to a significant improvement of patient survival in metastatic colorectal cancer (CRC) with DNA mismatch repair-deficiency (dMMR)/microsatellite instability-high (MSI-H). However, not all these patients are sensitive to monoimmunotherapy. We firstly presented a case series of advanced dMMR/MSI-H CRCs treating with PD-1 inhibitor-based chemoradioimmunotherapy (CRIT).

**Methods and Materials:**

We assessed the short-term efficacy and safety of CRIT in advanced dMMR/MSI-H CRCs, and also did next-generation sequencing (NGS) assays.

**Results:**

Our analysis included five advanced dMMR/MSI-H CRCs who have received toripalimab-based CRIT. Toripalimab was given 240mg every three weeks, and the radiation dose was 45-50 gray in 25 fractions. Chemotherapy regimens consisted of CAPOX in three patients, capecitabine in one patient, and mFOLFOX6 in one patient. Initially, two patients displayed complete response (CR), and three patients achieved partial response (PR) on imaging findings. Afterwards, one PR patient was confirmed pathological complete response after surgery, leading to three CR cases in total. Hematological toxicity was the most common adverse effect, and only two patients developed mild immune-related adverse effects besides. All the treatment-related adverse events were under control. Based on the NGS results, the median intratumor heterogeneity was 0.19 (range 0-0.957), which was less in CR patients than PR patients (*P* = 0.019). Genetic mutations at DNA damage repair genes and the *JAK1* gene were also observed.

**Conclusions:**

For advanced dMMR/MSI-H CRC, anti-PD-1 based CRIT is effective and safe. Further studies are required to better clarify the potential role and mechanism of CRIT as a viable therapeutic strategy in this population.

## Introduction

The latest cancer statistics showed that the incidence and mortality of colorectal cancer (CRC) ranked third among all cancer in men and women (1). By the year 2030, there will be more than 2.2 million new cases and 1.1 million deaths of CRC (2). In the past ten years, immunotherapy especially immune checkpoint blockade (ICB) therapy, has become a major therapeutic strategy for multiple types of solid cancers. ICB, including programmed cell death protein-1 (PD-1) inhibitors and anti-cytotoxic T lymphocyte antigen-4 (CTLA-4) antibodies, have significantly improved patients’ survival in DNA mismatch repair-deficiency (dMMR)/microsatellite instability-high (MSI-H) metastatic colorectal cancer (mCRC) (3–8). As a result, the FDA has approved PD-1 inhibitors, pembrolizumab and nivolumab, as the second-line therapy for dMMR/MSI-H mCRC. Nivolumab ± ipilimumab or pembrolizumab alone were recommended for neoadjuvant treatments for resectable or unresectable synchronous liver and/or lung metastastic dMMR/MSI-H CRC by the NCCN guideline (9, 10), and for primary treatments for unresectable metachronous metastatic dMMR/MSI-H CRC who has received FOLFOX/CAPOX therapy within past 12 months (9, 10). However, there are still a large amount of dMMR/MSI-H CRCs who are resistant to ICB. To date, many ongoing clinical trials of combined ICB with chemotherapy, targeted therapy, or radiotherapy aim to improve the efficacy of ICB in these patients. For instance, chemoradiotherapy can upregulate PD-L1 expression in rectal cancer (11). PD-1 or PD-L1 antibodies did not only improve local control but also had a systemic efficacy in irradiated colon cancer mouse models (12). Besides, a preclinical study also showed that chemotherapy combined with ICB could enhance radiotherapy-induced abscopal effects (13). Therefore, it can be probably inferred that chemoradioimmunotherapy (CRIT) may improve the efficacy of ICB in dMMR/MSI-H CRC patients. Here, we firstly presented a retrospective case series of five advanced dMMR/MSI-H CRC patients who achieved good response to CRIT.

## Materials and Methods

### Study Design

From March 2019 to December 2019, advanced dMMR/MSI-H CRC patients receiving CRIT were retrospectively analyzed in this study.

This study was conducted following the Declaration of Helsinki and received the full approval of the Sun Yat-sen University Cancer Center Institutional Review Board on Medical Ethics (B2020-141-01). All patients provided written informed consent before therapy.

### Next-Generation Sequencing (NGS)

Five formalin-fixed paraffin-embedded (FFPE) specimens of all patients were obtained before CRIT. All specimens were primary tumors except abdominal metastasis of patient 2.

Genomic DNAs were isolated from FFPE specimens and blood using the GeneRead DNA FFPE Kit (Qiagen) and Qiagen DNA blood mini kit (Qiagen), respectively. Then, extracted DNAs were amplified, purified, and analyzed using an NGS panel (YuceOneTM Plus X, Yucebio, China).

Sequencing reads with > 10% N rate and/or > 10% bases with quality score < 20 were filtered using SOAPnuke (Version 1.5.6). The clean reads were mapped to the UCSC reference human genome (version hg19) using the Burrows Wheeler Alignment tool (BWA, Version 0.7.12). SAMtools (Version 1.3) was used for alignment data conversion, sorting, and indexing. The duplicates were marked by SAMBLASTER (Version 0.1.22) to reduce biases in downstream analyses. The single nucleotide variants were detected using VarScan (Version 2.4) with parameters –min-coverage-normal 20 –min-coverage-tumor 20 –min-reads23. The mutations were filtered using a customized Perl script to eliminate false positives and annotated by SnpEff (Version 4.3).

Tumor mutational burden (TMB) was calculated using non-silent somatic mutations, including coding base substitution and indels. TMB > 20 muts/Mb was defined as TMB-High (TMB-H). Microsatellite instability (MSI) scores were analyzed by MSIsensor (Version 0.2). MSI scores > 20 were defined as MSI-High (MSI-H). Tumor neoantigen burden (TNB) was measured as the number of mutations that could generate neoantigens per megabase. TNB > 4.5 neos/Mb was defined as TNB-High (TNB-H). The ratio of subclone mutations to all mutations was interpreted as intratumor heterogeneity (ITH).

Frameshift, nonsense, and splice site alterations were classified as deleterious. Missense mutations reported as pathogenic by the Catalogue of Somatic Mutations in Cancer (COSMIC) (14) and/or ClinVar (15) databases, and/or with a SIFT score of <0.05 (16), were classified as deleterious.

### Clinical Evaluation

Pretreatment tumors were staged according to the criteria of the American Joint Committee on Cancer 8th edition. Clinical response based imaging findings was assessed according to the RECIST 1.1 (17). For surgical specimens, no residual tumor cell was defined as pathological complete response (pCR). Follow-up data were collected from the follow-up platform of the hospital.

### Statistical Analysis

Statistical analyses were performed using SPSS 26.0 statistical software (IBM, NY, USA). Comparisons between two groups were evaluated by Student t-test. *p* < 0.05 at two sides was considered statistically significant.

## Results

### Patient Demographic and Clinical Characteristics

**Figure 1:**
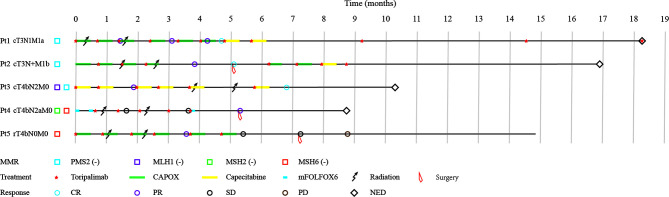
Patient characteristics, treatments, and outcomes. CR, complete response; PD, progressive disease; PR, partial response; SD, stable disease.

Overall, five dMMR/MSI-H CRC patients were identified and enrolled. The median age was 37 years old (range 27-64), and three (3/5) were male. Of all patients, primary tumors were located at right colon (n=3), left colon (n=1) and rectum (n=1). All patients were adenocarcinoma except one adenosquamous carcinoma. Two (2/5) patients had metastases: right kidney metastasis in patient 1, and liver and abdominal cavity metastases in patient 2. The other three patients had large tumors invaded adjacent organs and regions, including the gallbladder, duodenum, liver, and peritoneum of patient 3, peritoneum and abdominal wall of patient 4, and psoas major muscle and ureter of patient 5. Prior to this study, patient 4 had received three cycles of CAPOX, but no tumor regression. Besides, patient 5 had received surgery and adjuvant FOLFOX chemotherapy for the primary tumor but found the tumor regrowth a year later. The demographic, clinical, and therapeutic details of the patients were shown in **Table 1** and **Figure 1**.

**Table 1:** Demographic features, clinical characteristics, and therapeutic regimens.

Items	Patient 1	Patient 2	Patient 3	Patient 4	Patient 5
Gender	Male	Male	Female	Female	Male
Age (years)	37	27	62	35	64
Tumor site	Rectum	Right colon	Right colon	Left colon	Right colon
Histology	Adenocarcinoma	Adenocarcinoma	Adenosquamous carcinoma	Adenocarcinoma	Adenocarcinoma
Stage	cT3N1M1a	cT3N+M1b	cT4bN2M0	cT4bN2aM0	rT4bN0M0
Metastatic site	Right kidney	Liver and abdominal cavity	None	None	None
Invaded adjacent organ	NA	NA	Gallbladder, duodenum, liver, and peritoneum	Peritoneum and abdominal wall	Psoas major muscle, and ureter
MSH2	+	+	+	–	+
MSH6	+	+	+	–	–
PMS2	–	–	–	+	+
MLH1	+	+	–	+	+
Previous chemotherapy	No	No	No	CAPOX	FOLFOX
Previous surgery	No	No	No	No	Radical surgery
Combined chemotherapy	CAPOX	CAPOX	Capecitabine	mFOLFOX6	CAPOX
Combined radiotherapy	50Gy/25F	50Gy/25F	45Gy/25F	48Gy/25F	50Gy/25F
Surgery after ICB	No	Radical surgery	No	Radical surgery	Palliative surgery
Total course of ICB	10	7	6	5	6
Course of ICB for best response	6	3	6	5	4
Imaging response	CR	PR	CR	PR	PR
Pathology response	NA	pCR	NA	TRG(2ypT4bN0M0)	NA

CR, complete response; ICB, immune checkpoint blockade; NA, not available; pCR, pathological complete response; PR, partial response; TRG, tumor regression response.

### Treatment Strategies

All patients received PD-1 inhibitor-Toripalimab 240 mg intravenously once every three weeks with a median number of 6 cycles (range 5-11 cycles) and a median treatment duration of 5.8 months (range 3.1-18.3 months). Each individualized chemotherapy regimen was decided according to patients’ previous treatment and health conditions. Patient 3 received capecitabine as she was old and weak. The chemotherapy regime of patient 4 was switched to mFOLFOX6 because she was insensitive to CAPOX. Other patients all received CAPOX. All patients were also treated with intensity-modulated radiation therapy (IMRT) for the primary tumor at 45-50 gray in 25 fractions. After CRIT, patient 2 and patient 4 underwent radical surgery, whereas patient 5 underwent palliative surgery. The integral treatments for each patient were illustrated in **Table 1** and **Figure 1**.

### Efficacy Results

After a median period of 4.4 months (range 3.1-6.8 weeks) from the first Toripalimab injection and 7.1 weeks (range 5.3-13.3 weeks) from the last IMRT, five patients all achieved objective responses, including two complete response (CR) and three partial responses (PR) according to RECIST 1.1. The two CR patients took a watch-and-wait strategy without surgery. Primary and metastases of patient 2 were confirmed pCR after surgery, resulting in three CR patients in total. Patient 4 underwent surgery and had a downstage tumor from T4bN2a to T4bN0, and the tumor regression grade was TRG2 based on NCCN guidelines. The primary tumor of patient 5 was smaller than the initial volume but found invaded the ureter, small intestine, and iliac blood vessels during surgery. As a result, patient 5 underwent palliative surgery for organ conservations. The images of all patients before and after CRIT were displayed in **Supplementary Figures 1–5**. With a median follow-up time of 14.8 months (range 8.1-18.3), only patient 5 had progressive disease at 8.8 months after the first Toripalimab injection. All the other cases had no clinical signs of disease progression or recurrence.

### Treatment-Related Adverse Events (TRAEs)

Till September 2020, all TRAEs happened for all patients were shown **Table 2**. The most common adverse events were leukopenia and neutropenia. Most TRAEs in this study were at grade 1 or grade 2. Only one patient experienced grade 4 thrombocytopenia. One patient had grade 2 immune-related increased TSH, and therefore had levothyroxine therapy. All the adverse events were under control, and patients recovered during treatment.

**Table 2:** Treatment-related adverse events.

Adverse event	Grade 1 (n)	Grade 2 (n)	Grade 3 (n)	Grade 4 (n)
Any	4	5	3	1
Leukopenia	1	2	1	0
Neutropenia	3	0	1	0
Anemia	1	1	1	0
Thrombocytopenia	0	0	1	1
ALT elevation	0	2	0	0
AST elevation	3	0	0	0
Increased creatinine	1	0	0	0
Diarrhea	0	2	0	0
Nausea	0	1	0	0
Vomit	0	1	0	0
Rash	1	0	0	0
TSH increased	0	1	0	0
fT3 decreased	0	1	0	0
Infection	0	2	0	0

ALT, alanine aminotransferase; AST, aspartate aminotransferase; TSH, thyroid stimulating hormone; fT3, free triiodothyronine.

### Genetic Analyses

**Figure 2:**
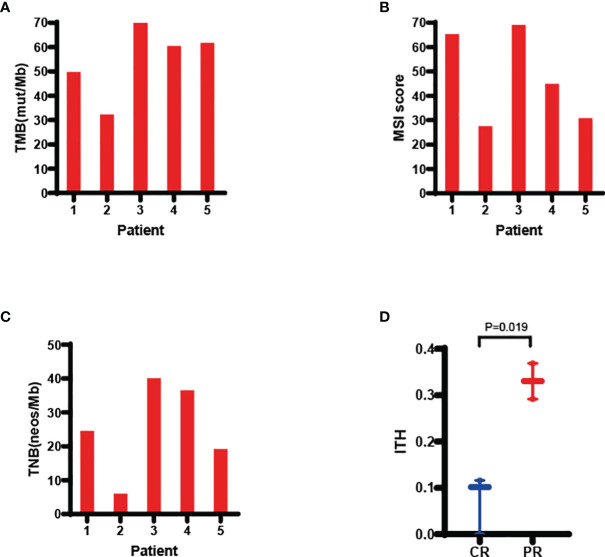
Molecular analyses of TMB **(A)**, MSI score **(B)**, TNB **(C)**, and ITH **(D)**. ITH was lower in CR than PR patients. Values were presented as median and range. Comparisons between CR and PR patients was tested using two-sided t-test. CR, complete response; CRIT, chemoradioimmunotherapy; ITH, intratumor heterogeneity; MSI, microsatellite instability; PR, partial response; TMB, tumor mutational burden; TNB, tumor neoantigen burden.

Based on NGS results shown in **Figures 2A–D**, the median value of TMB, MSI score, TNB and ITH were 60.48mut/Mb (range 32.31-77.18), 44.95 (range 27.45-69.06), 24.56 neos/Mb (range 5.98-40.09) and 0.116 (range 0-0.369), respectively. CR patients had a lower ITH than PR patients (*p*=0.019, **Figure 2D**). TMB, MSI scores, and TNB did not correlate with treatment response (**Supplementary Figures 6A–C**).

Additionally, 19 mutations in 12 DNA damage repair (DDR) genes were observed. Among them, most alternations were missense mutations (10/19, 52.6%), the remaining included frameshift mutations (6/19, 31.6%), splice site (2/19, 10.5%), and nonsense alterations (1/19, 5.3%). *ATM* (4/5, 80.0%) was the most commonly mutated DDR gene. All missense mutations were unique, of which only 7 were previously reported in COSMIC and/or ClinVar database and 5 of them were deleterious. In silico evaluation, another 2 potentially deleterious missense mutations were identified. The median number of deleterious DDR mutations patients harbored was 3 (range 1-6). All the DDR gene mutations were listed in **Supplementary Table 1**. Genes involved in the mismatch repair pathway (6/10, 60%) were the most common (**Supplementary Table 2**).

Furthermore, *JAK1* deleterious mutation was observed in patients 1 (K924fs) and 3 (T533M), and *JAK1* neutral mutation was found in patient 4 (R93H). The most frequently altered genes were *APC* (100%), *FAT1* (80%), *TRRAP* (80%), *KAT6B* (80%), *CIC* (80%) and *ATM* (80%) (**Supplementary Figure 7**).

## Discussion

In this study, we firstly reported a retrospective case series of advanced dMMR/MSI-H CRC patients treated with CRIT. After treatment, all five patients achieved beneficial responses of pCR (1/5, 20%), cCR (2/5, 40%), or PR (2/5, 40%), leading to a 100% overall response rate. The good efficacy suggested that this strategy might be a promising treatment for advanced dMMR/MSI-H CRC.

It has been well studied that locally advanced rectal cancer can benefit from neoadjuvant chemoradiotherapy. Our previous work found that locally advanced unresectable colon cancer responded well to neoadjuvant chemoradiotherapy, with a 26.3%-38.1% pCR rate (18, 19). Consequently, neoadjuvant chemoradiotherapy has been recommended for T4b patients with local invasion of the sigmoid colon by the Chinese Society of Clinical Oncology (20). However, whether dMMR/MSI-H CRC patients could benefit from preoperative chemoradiotherapy as well as microsatellite instability stale or low CRC was still a controversial clinical question (21, 22). In our present cohort, a 100% objective response rate and 60% CR rate were achieved, indicating that they could benefit from CRIT.

For patients whose primary tumor did not invade adjacent organs, immunotherapy alone or combined with chemotherapy could result in CR (23, 24). So, radiotherapy and chemotherapy may not be essential for these patients. But, the two T3 patients with metastases were treated with CRIT to maximize the abscopal effect, and they finally achieved CR. Similarly, a preclinical study also showed that the abscopal tumor has the best response to triple therapy of cisplatin, radiation, and PD-1 inhibitor (13), implying that CRIT is effective for mCRC.

Though all patients in our study were effective, two patients with T4b colon cancer did not achieve CR. One of them was still unresectable after CRIT, and his disease progressed 8.8 months after the Toripalimab initiation. In the NICHE study (23), only one patient with T4b tumor and three of seven patients with T4a tumors did not achieve pCR. Also, some T4b tumors could not achieve CR after neoadjuvant PD-1 blockade in published case reports (24, 25). For dMMR/MSI-H CRC with large tumors invading multiple essential organs, the clinical response to single-agent immunotherapy or immunotherapy-based combination therapy is still unsatisfactory. Thus, it is necessary to explore optimized regimens of these therapeutic methods to achieve better clinical efficacy in these patients.

Up to now, DDR gene alterations, especially deleterious alternations, have been demonstrated to be associated with improved clinical outcomes in metastatic urothelial cancer (26), metastatic clear cell renal cell carcinoma (27), and non-small-cell lung cancer (28) treated with PD-(L)1 inhibitors. Likewise, a recent study also found that CRC patients with DDR mutations could obtain a better prognosis when using ICB (29). This was consistent with our patients that DDR genes alterations were frequent, which may contribute to their effective response to CRIT. Additionally, *ATM* was the most commonly mutated DDR gene in these CRC and patients with *ATM* mutations had a significantly better overall survival than those without when treated with ICB (30). In addition, *ATM* inhibitors potentiated anti-PD-1 therapy in the mouse model (30). It was also reported that *ATM* inhibition and radiation could enhance the efficacy of ICB by increasing tumoral immunogenicity (31). Therefore, *ATM* may be a potential biomarker of immunotherapy, and *ATM* inhibitors with ICB and radiation may be an efficacious therapeutic regimen. Interestingly, *ATM*, *ATR*, and *LIG3* genes co-mutation was only found in two PR patients. This might imply that the molecular contributions of multiple DDR gene co-mutation may differ from single DDR gene mutation. Further studies are needed to disclose the effects and mechanisms of how DDR mutations impact sensitivity to CRIT.

Additionally, driver mutations of the JAK-STAT pathway may contribute to tumor progression during immunotherapy (32, 33). In our study, both of the two patients with pathogenic *JAK1* mutation achieved CR, and the patient with neutral *JAK1* mutation achieved PR. Perhaps, chemoradiotherapy can dismiss the negative impact of *JAK1* mutations in some way.

There were some limitations to our study. Our study only enrolled five patients, and the follow-up period was short. Also, baseline characteristics and treatment strategies were not incoherent of all patients. Although we did genetic analysis, we could not illustrate the definite mechanism of CRIT. Consequently, our observations warrant further considerations and validations in a larger sample size. We are doing a phase II clinical study (NCT04301557) of Toripalimab combined with chemoradiotherapy for dMMR/MSI-H locally advanced CRC, which may provide more clinical evidence to clarify the role of CRIT for CRC.

In general, CRIT is effective and safe for advanced dMMR/MSI-H CRC. Further studies are required to investigate the potential role and mechanism of CRIT in this population.

## Data Availability Statement

The datasets presented in this study can be found in online repositories. The whole sequence data reported in this paper available in the Genome Warehouse in National Genomics Data Center, Beijing Institute of Genomics, Chinese Academy of Sciences, under accession number PRJCA007192 at https://ngdc.cncb.ac.cn/databases, upon reasonable request.

## Ethics Statement

The studies involving human participants were reviewed and approved by Sun Yat-sen University Cancer Center Institutional Review Board on Medical Ethics. The patients/participants provided their written informed consent to participate in this study.

## Author Contributions 

Conceptualization: CJZ, TJ, and WWX. Methodology: CJZ, WWX, and YJX. Data curation: CJZ, QXW, and PQC. Investigation: YTZ, ZKZ, DFW, and HQL. Resources: ZFZ, RZ, and YHG. Software: CJZ and WWX. Writing - Original draft: CJZ, WWX, and YJX. Editing: CS. Writing-Review and Editing: RZ and YHG. All authors contributed to the article and approved the submitted version.

## Funding

This work was supported by the National Natural Science Foundation of China under Grant 81672987 and 82073329; and the Natural Science Foundation of Guangdong Province under Grant 2020A1515011286.

## Conflict of Interest

Authors YJX, YTZ, ZKZ, DFW, HQL and CS are employed by YuceBio Technology Co., Ltd.

The remaining authors declare that the research was conducted in the absence of any commercial or financial relationships that could be construed as a potential conflict of interest.

The reviewer MYC declared a shared affiliation with several of the authors (CJZ, TJ, QXW, ZFZ, PQC, RZ, WWX and YHG) to the handling editor at time of review.

## Publisher’s Note

All claims expressed in this article are solely those of the authors and do not necessarily represent those of their affiliated organizations, or those of the publisher, the editors and the reviewers. Any product that may be evaluated in this article, or claim that may be made by its manufacturer, is not guaranteed or endorsed by the publisher.

## Abbreviations

CRC, colorectal cancer; dMMR, mismatch repair-deficiency; MSI-H, microsatellite instability-high; CRIT, chemoradioimmunotherapy; PD-1, programmed cell death protein-1; CR, complete response; PR, partial response; pCR, pathological complete response; ICB, immune checkpoint blockade; CTLA-4, cytotoxic T lymphocyte antigen-4; mCRC, metastatic colorectal cancer; FFPE, formalin-fixed paraffin-embedded; TMB, tumor mutational burden; TNB, tumor neoantigen burden; ITH, intratumor heterogeneity; COSMIC, the Catalogue of Somatic Mutations in Cancer; TRAE, treatment-related adverse events; DDR, DNA damage repair.
