# Supplementary material for: Good Tumor Response to Chemoradioimmunotherapy in dMMR/MSI-H Advanced Colorectal Cancer: A Case Series
Source: Front Immunol. 2021 Dec 15;12:784336. doi: 10.3389/fimmu.2021.784336 (PMC8714781; doi:10.3389/fimmu.2021.784336)
Supplement: Supplementary file 1 [file DataSheet_1.docx]

**Supplementary materials**

**Good Tumor Response to** **Chemoradioimmunotherapy in dMMR/MSI-H Unresectable Advanced Colorectal Cancer: A Case Series**

*Cheng-jing Zhou, Wei-wei Xiao**, Ya-jie Xiao, Qiao-xuan Wang, Zhi-fan Zeng, Pei-qiang Cai, Yong-tian Zhao, Zhi-kun Zhao, Dong-fang Wu, Han-qing Lin, Rong Zhang, Yuan-hong Gao*

**Supplementary Table 1.** DNA damage repair response genes alternations detected.

| Patient No. | Gene | AA change | Type of alterations | Proportion (%) | Deleterious |
| --- | --- | --- | --- | --- | --- |
| 1 | ATM | S214fs | Frameshift mutation | 33.26 | Yes |
| 1 | BRCA2 | A2851V | Missense mutation | 32.00 | Yes |
| 1 | MLH1 | Splice variant | Splice site mutation | 27.70 | Yes |
| 1 | MSH6 | F1088fs | Frameshift mutation | 24.96 | Yes |
| 1 | POLE | A81V | Missense mutation | 2.09 | No |
| 2 | MSH6 | R1095H | Missense mutation | 8.27 | Yes |
| 3 | ATM | G52* | Nonsense mutation | 21.88 | Yes |
| 3 | MSH3 | N793D | Missense mutation | 26.45 | No |
| 3 | PRKDC | A3899V | Missense mutation | 26.65 | Yes |
| 4 | ATM | Q2762fs | Frameshift mutation | 4.17 | Yes |
| 4 | ATR | R1814fs | Frameshift mutation | 9.56 | Yes |
| 4 | LIG3 | V473I | Missense mutation | 8.81 | Yes |
| 4 | MLH1 | P654S | Missense mutation | 8.49 | Yes |
| 4 | MLH3 | R381S | Missense mutation | 11.86 | No |
| 4 | MSH2 | G683R | Missense mutation | 12.28 | Yes |
| 4 | PMS1 | C165fs | Frameshift mutation | 3.16 | Yes |
| 5 | ATM | Splice variant | Splice site mutation | 9.82 | Yes |
| 5 | ATR | L1081P | Missense mutation | 9.66 | Yes |
| 5 | LIG3 | S241fs | Frameshift mutation | 11.76 | Yes |

**Supplementary Table 2.** Relevant genes in DNA damage repair response (DDR) pathways.

| Pathway | Genes |
| --- | --- |
| Mismatch repair | MLH1, MLH3, MSH2, MSH3, MSH6, PMS1 |
| Base excision repair | LIG3, POLE |
| Checkpoint factor | ATM, ATR |
| Fanconi anemia | BRCA2 |
| Non-homologous end joining | BRCA2 |
| Homologous recombination repair | PRKDC |


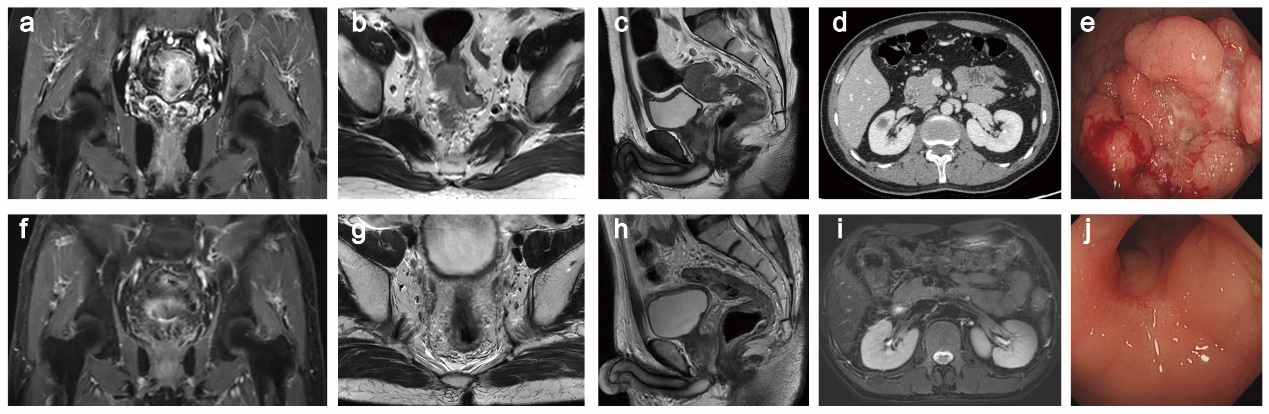
**Supplementary Figure 1.** **Imaging of patient 1 before and after CRIT.** (a-c) Images of the primary rectal tumor before CRIT. (d) Image of the right renal metastasis before CRIT. (e) Rectal tumor mass at endoscopy before CRIT. (f-h) No residual rectal tumor after CRIT. (i) No residual right renal metastasis after CRIT. (e) No residual rectal tumor at endoscopy after CRIT. CRIT, chemoradioimmunotherapy.


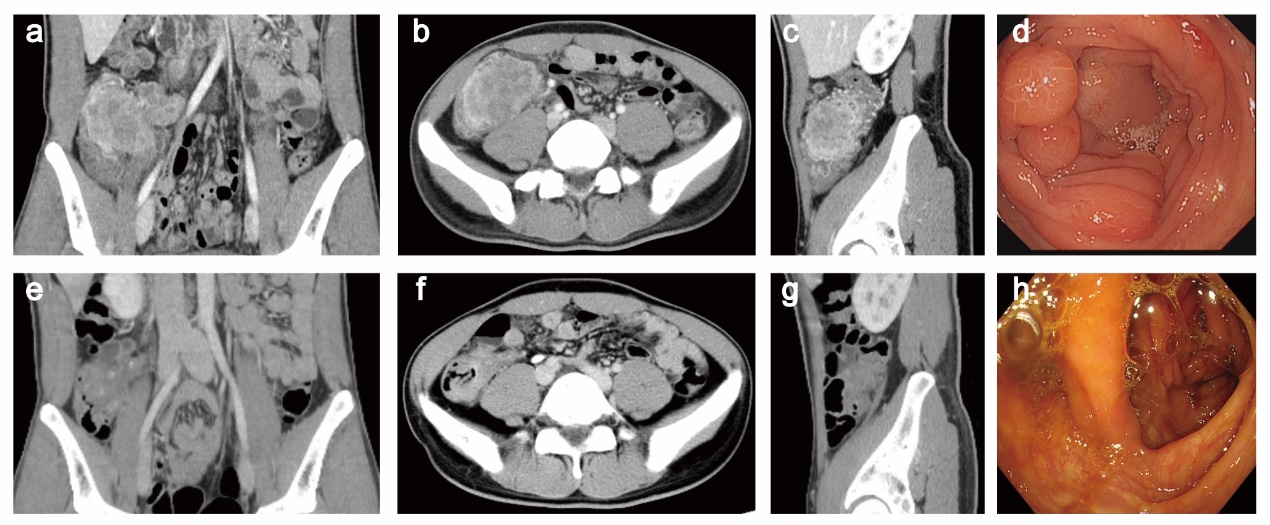
**Supplementary Figure 2.** **Imaging of patient 2 before and after CRIT.** (a-c) Images of the primary right colon tumor before CRIT. (d) Right colon tumor mass at endoscopy before CRIT. (e-g) No residual right colon tumor after CRIT. (h) No residual tumor at endoscopy after CRIT. CRIT, chemoradioimmunotherapy.


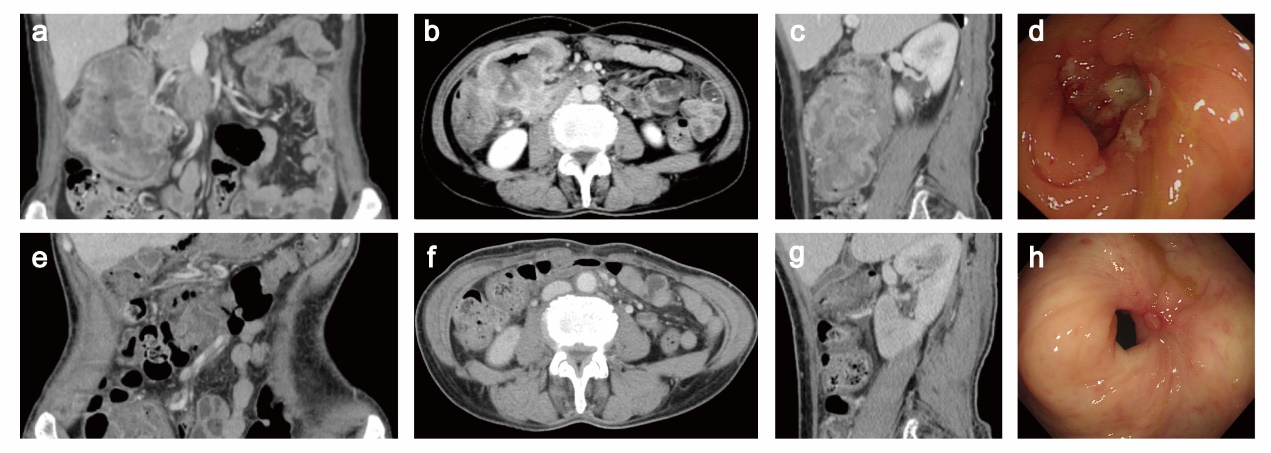
**Supplementary Figure 3.** **Imaging of patient 3 before and after CRIT.** (a-c) Images of the primary right colon tumor before CRIT. (d) Right colon tumor mass at endoscopy before CRIT. (e-g) No residual right colon tumor after CRIT. (h) No residual tumor at endoscopy after CRIT. CRIT, chemoradioimmunotherapy.


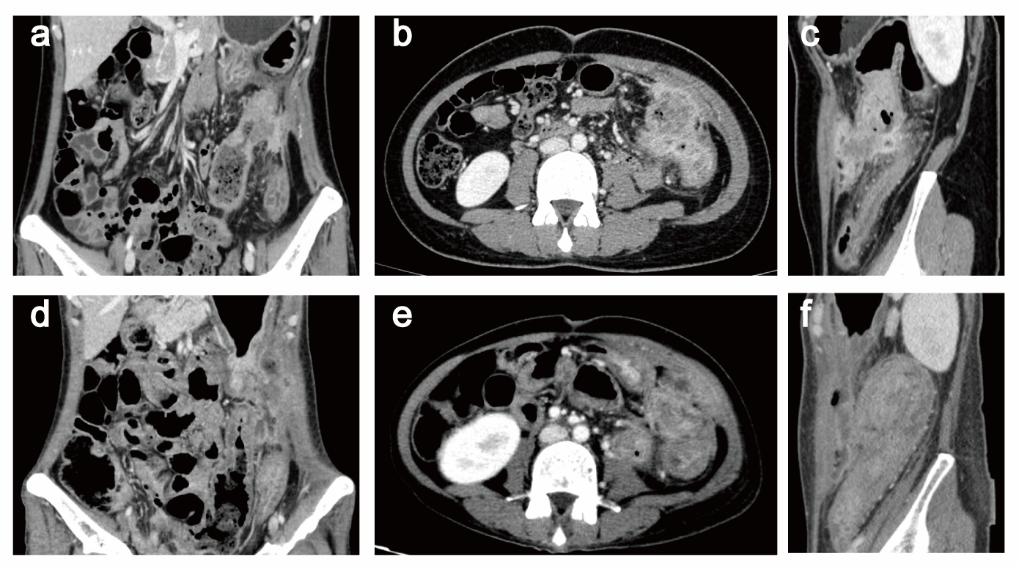


**Supplementary Figure 4.** **Imaging of patient 4 before and after CRIT.** (a-c) Images of the primary left colon tumor before CRIT. (d-f) Images of the partial regression left colon tumor after CRIT. CRIT, chemoradioimmunotherapy.


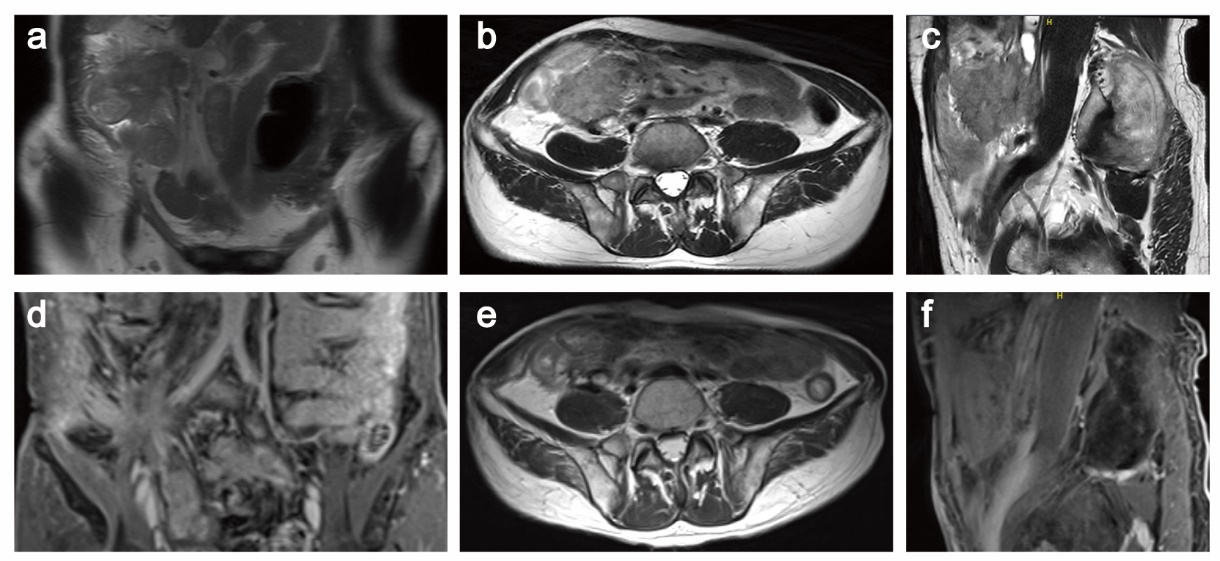
**Supplementary Figure 5.** **Imaging of patient 5 before and after chemoradioimmunotherapy.** (a-c) Images of the primary right colon tumor before CRIT. (d-f) Images of the partial regression right colon tumor after CRIT. CRIT, chemoradioimmunotherapy.


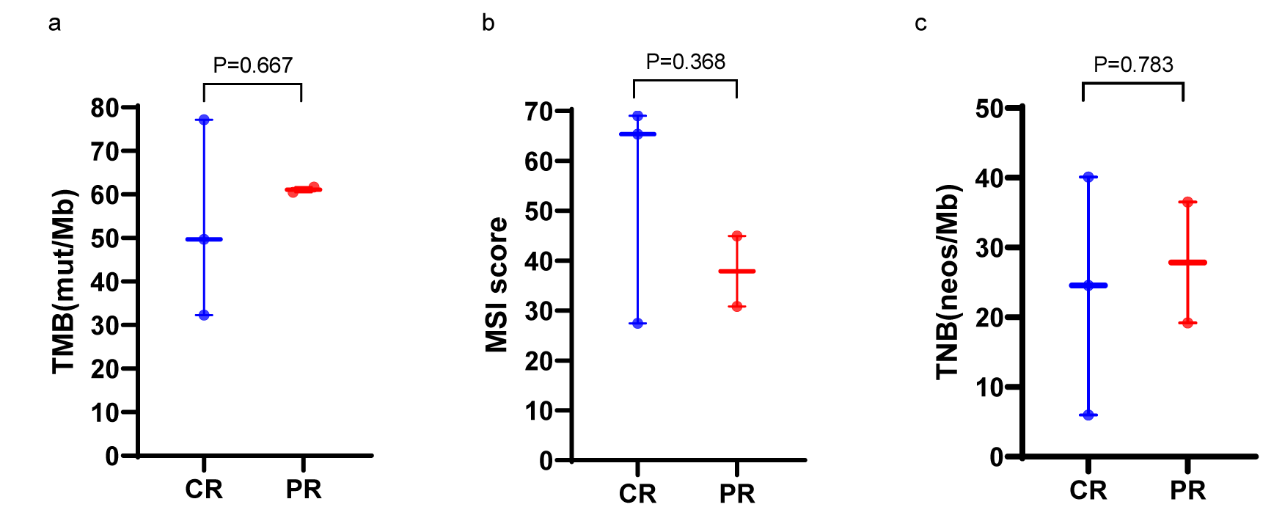
**Supplementary Figure 6.** **Molecular analyses between CR and PR patients.** TMB (a), MSI score (b), and TNB (c) have no significant differences between CR and PR patients. Values were presented as median and range. Comparisons between CR and PR patients were tested using Student t-test. Statistical tests were. CR, complete response; PR, partial response.


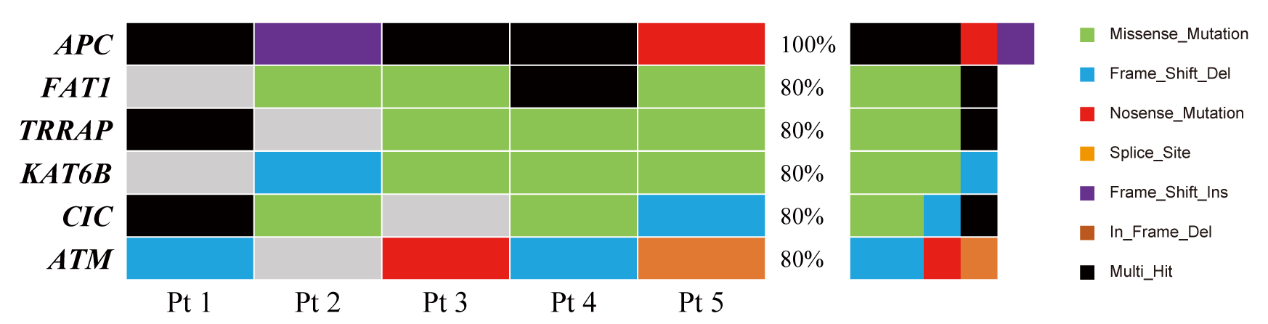


**Supplementary Figure 7. The most frequently altered genes.** *APC* (100%), *FAT1* (80%), *TRRAP* (80%), *KAT6B* (80%), *CIC* (80%) and *ATM* (80%) were the most frequently altered genes in our study patients. Pt, patient.
